# Factors Associated with Physical Activity Increases and Decreases Among a Sample of Appalachian Residents During the COVID-19 Pandemic: A Cross-Sectional Study

**DOI:** 10.13023/jah.0501.04

**Published:** 2023-04-01

**Authors:** Christiaan G. Abildso, Angela M. Dyer, Shay M. Daily, Christa Lilly, Emily A. Sarkees, Samantha I. Moyers, Thomas K. Bias

**Affiliations:** West Virginia University School of Public Health, cgabildso@hsc.wvu.edu; West Virginia University Office of Health Affairs; West Virginia University Office of Health Affairs; West Virginia University School of Public Health

**Keywords:** Appalachia, Community Health Needs Assessment, COVID-19, physical activity, rural

## Abstract

**Introduction:**

Physical activity (PA) can prevent and reduce the deleterious physical and mental health effects of COVID-19 and associated lockdowns. Research conducted early in the pandemic demonstrates that a greater proportion of adults in the U.S. have decreased than increased PA, and the effects vary by sociodemographic factors. Ongoing evidence is important to identify patterns in PA changes during the pandemic.

**Purpose:**

This study aims to identify factors associated with increases and decreases in PA during the COVID-19 pandemic in a convenience sample of adults residing in Appalachia. Methods: Surveys were collected from a convenience sample of adults from eight counties in West Virginia from January to March 2021. Logistic regression analysis was used to identify sociodemographic, health, and rurality factors associated with (1) increased PA and (2) decreased PA during the pandemic, assessed retrospectively via self-report.

**Results:**

Analysis of 1,401 survey responses revealed that better self-rated health, lower body mass index, and higher income and education were associated with a greater likelihood of more time spent doing PA during the pandemic ( *p* ≤ .05). Respondents with lower self-rated health, higher body mass index, lower income, and lower levels of education—plus females and those living in a more urban county—were more likely to spend less time doing PA during the pandemic ( *p* ≤ .05).

**Implications:**

Analyses suggest that pre-pandemic disparities in PA by health, wealth, and education were exacerbated during the pandemic. These must be addressed before physical inactivity and ill health become endemic to the Appalachian Region.

## INTRODUCTION

A multitude of disease morbidity and mortality indicators are worse in Appalachia than in the rest of the U.S.^1^ Nearly all the indicators (e.g., cardiovascular disease, type 2 diabetes) are associated with physical inactivity,^2^,^3^ which is also significantly higher in Appalachia.^1^ Sufficient amounts of physical activity (PA)—a behavior with distinct etiology and effects from those of physical inactivity^4^—is essential to the prevention and treatment of these diseases. Notably, research has also linked PA to the reduction of negative physical and mental health impacts of COVID-19. Physical health benefits include reduced susceptibility to and severity of COVID-19 outcomes^5^ by strengthening immune function, preventing chronic disease, and reducing inflammation.^6^ PA is also beneficial in the prevention and treatment of anxiety, depression, and other mental health concerns exacerbated by pandemic isolation.^7^ PA has demonstrated associations with well-being by helping people experience detachment, relaxation, mastery experiences, and control of leisure time during COVID-19-related lockdowns.^8^

Several studies evaluating changes in PA among adults in the U.S. during the early months of the pandemic demonstrate that PA declined in spring and summer 2020 immediately after the pandemic was declared^9^–^11^ and remained at the reduced level in spring and early summer 2021.^12^ Dunton et al.’s analyses of survey data^9^ collected between April 19 and May 25, 2020, from 268 respondents (58% of whom lived in California) demonstrated significant decreases of 60–90 minutes per week of moderate-intensity PA, vigorous-intensity PA, and walking. Self-report data from 1,809 respondents (65.6% residing in Texas) collected from April 15 to May 5, 2020, demonstrated that more respondents decreased their PA than increased (39.0% and 25.2%, respectively) since the pandemic began.^10^ Using a similar single-item assessment of PA change among 3,829 U.S. respondents to national consumer panel surveys from March 19 to April 9, 2020, and June 10 to 25, 2020, Watson et al.^11^ also found that more respondents reported PA decreases than increases (30.4% and 20.3%, respectively). The pattern of findings was supported by analysis of step count data from smartphones in a subset of 143 of the respondents in the Dunton et al. study^9^ that showed an average reduction of 2,232.4 steps per day (April 15 to May 5, 2020), and from the 10 U.S. cities in Tison et al.’s global study^13^ that showed roughly 20–40% decreases in daily step counts in mid-April from baseline (February).

There have been sociodemographic and geographic disparities in COVID-19-related PA changes. For instance, Dunton et al.^9^ discovered greater decreases in self-reported minutes of walking from April 15 to May 5, 2020, for participants who were Hispanic and low-income, while Watson et al.^11^ found that reporting being *more active* from March/April to June 2020 was associated with being female, aged 18–44 years, White, not obese, educated at or above college level, residing in urban areas and in the Midwest region. Knell et al.^10^ confirmed the finding that females were more likely to increase PA and found that people with children in the home were more likely to increase PA from April 15 to May 5, 2020. These results align mostly with pre-pandemic PA trends,^14^ apart from the disparity between the sexes. Pre-existing disparities in access to safe places to be active by rural–urban status^15^ and by socioeconomic status^16^ may have exacerbated the pandemic-related PA changes. Surveys conducted during the pandemic reveal that at least half of the respondents are active at home or on roads/sidewalks in their neighborhoods.^9^,^11^ Home exercise equipment and walkable neighborhood spaces are less common in low-income, rural areas^15^ like much of Appalachia.

There is also evidence from 10 cities in the U.S.^13^ that PA declines witnessed during the early, most restrictive lockdown phases of the pandemic (February to mid-April, 2020), may abate toward pre-pandemic levels over time, but may lag behind the easing of COVID-19-related policy restrictions.^12^ To date, there is no evidence of PA changes during the COVID-19 pandemic experienced by residents of Appalachia. To better our understanding of the impact of the pandemic on PA over time in the U.S., ongoing research evaluating pandemic-related PA changes and disparities by geography and sociodemographic characteristics is needed. Thus, this study assesses the sociodemographic and geographic characteristics associated with increases and decreases in PA during the pandemic in a sample of Appalachian adults using a retrospective, cross-sectional survey.

## METHODS

### Data Collection

Data for this study were collected from January to March 2021 as part of the community input process of the Community Health Needs Assessments (CHNAs) for two non-profit hospitals in West Virginia (WV) with service areas covering eight contiguous counties in Western WV (see Table 1). The community served was defined by the CHNA leadership team for Hospital 2 (H2) as only one county (Jackson), whereas the team for Hospital 1 (H1) chose the seven other counties that are listed in Table 1. As an Internal Revenue Service requirement, at least every three years all non-profit hospitals in the U.S. are required to conduct a CHNA to inform decision-making. CHNAs provide a unique opportunity to collect health behavior and health status data to overcome the lack of time-sensitive data in areas of limited population that are often underserved by national surveillance data.

A convenience sample of survey responses was collected using a snowball sampling technique. Hospital staff and community partners were provided an anonymous link to an online survey^17^ and asked to share the link with personal and professional contacts by email. Additionally, H1 utilized the inbox/messaging capabilities within their electronic medical records system to send the survey link to patients within their seven-county service area and shared it via their social media accounts. Both hospitals also worked with community partners (e.g., United Way, public libraries) to disseminate paper copies of the survey to capture responses from populations under-represented in electronic health records and online surveys. Completed paper surveys were returned to the research team for input via the anonymous link. Individuals under age 18 years or residing outside a county served by the hospital were excluded from completing a survey. No incentives were provided to survey respondents. The study was approved for protection of human subjects by the West Virginia University Institutional Review Board (protocol no. 2003942005).

### Measures

The surveys included sections of items about community health perceptions and priorities, healthcare access, individual health behaviors, and COVID-19-related health behavior changes.

#### Dependent Variables

Two dependent variables were used in analyses: (1) increased PA during the pandemic and (2) decreased PA during the pandemic. Survey respondents were asked, “In the past six months, which of the following are things you have done in response to the coronavirus pandemic?” Respondents selected all that applied from a list of 24 response options, with 23 pandemic-related behaviors and a “None of the above” response option. The multiselect item technique and the list of pandemic-related impacts is an amalgam of what has been used in national studies in the U.S. (i.e., the COVID-19 Participant Experience Survey [COPE] and the COVID Impact Survey).^18^,^19^ In effect, each item in the list is treated as separate survey items with a binary response: “yes” if checked and “no” if not checked. Thus, there were 23 items, two of which were utilized for dependent variables: “Spent more time doing physical activity” and “Spent less time doing physical activity.”

If a respondent selected “Spent more time doing physical activity,” that response was coded as 1 for “yes” for increased PA. Similarly, if the respondent selected “Spent less time doing physical activity”, that response was coded as 1 for “yes” for decreased PA. The method for determining the “no” response for each of the dependent variables required a review of the full set of 24 response options to determine whether the respondent skipped the question or truly did not select the item (i.e., a “no” response). For each dependent variable, if the respondent did not select the PA option but did select at least one of the other pandemic-related behaviors (i.e., did not skip the question), that response was coded as 0 (“no”) for the dependent variable. To be conservative, if respondents selected both PA options (i.e., both increased and decreased PA in response to the pandemic); none of the 24 items; or “None of the above”, then those responses were coded as missing for both dependent variables and excluded from analyses.

A state of emergency was in place in WV during the entire data collection period, inclusive of the item-response recall period of “in the past 6 months” (i.e., July 2020 to March 2021). COVID-19 restrictions in place or removed are noted as follows based on the Governor’s executive orders.^20^ On July 1, 2020, several outdoor activities were permitted to resume, including fairs, festivals, amusement parks, and outdoor open-air concerts. However, this was rescinded two weeks later, and a limit of social gatherings to 25 people was put in place due to a spike in COVID-19 cases. In September, that was reduced to 10 based on the degree of community spread of COVID-19, which was tracked weekly using a color-coded system that also determined whether in-person K–12 instruction and scholastic sports could occur. From September 8, 2020, until the December holidays break, school systems varied in delivery based on parents’ choice to send their child to school full-time in person, virtually, or two days per week in person (“hybrid”). The first vaccinations occurred on December 14, 2020. The majority of K–12 school systems (48 of 55) returned to in person instruction 5 days per week on January 19, 2021, but winter sports were not allowed to resume until March. Outdoor fairs, festivals, and recreation-related businesses were allowed to reopen, and restaurants and bars were allowed to increase capacity from 50% to 75% in late February. On March 5, 2021, restaurants and bars were allowed to increase to full capacity and K–12 winter sports were allowed to resume unless an outbreak was indicated using the color-coded tracking system. On March 24, 2021, the use of the color-coded tracking system ended for all schools and related activities, and all live, indoor music performances were allowed to resume.

#### Independent Variables

Independent sociodemographic variables included age (*18–44, 45–64, ≥65 years [referent]*), self-rated health (*good/excellent, very poor/poor/fair [referent])*, body mass index (BMI) using self-reported height and weight (*underweight/normal weight, overweight, obese [referent]*), sex (*male, female [referent]*), marital status (*married/in a domestic partnership, all others [referent]*), ethnicity (*not Hispanic or Latino, Hispanic or Latino*), race (*White, all other races [referent]*), yearly household income ( *≥$75,000; $50,000 to $74,999; $30,000 to $49,999; < $30,000 [referent]*), education (*bachelor’s degree or higher, completed some post-secondary education, ≤ High school diploma or equivalent (GED) [referent]*), and children in the home (*none, one or more [referent]*). County-level rurality (*mostly urban*, *mostly rural*, *completely rural [referent])* was determined by coding each respondent’s self-reported county of residence based on the percentage of a county’s population not living in an urban area (see Table 1) according to 2010 Census data.^21^ Counties with <50% of the population living in rural areas are classified as *mostly urban*; 50 to 99.9% as *mostly rural*; 100% rural as *completely rural*. Previous research has shown variation in county-level environmental factors associated with adults’ PA along the rural–urban continuum when using this variable.^22^ County-level rural–urban categorizations based on population and commuting data (e.g., Rural-Urban Continuum Codes, Urban Influence Codes) were not used, as commuting was severely restricted during the data collection period due to the COVID-19 restrictions.

### Statistical Methods

Analyses were conducted using SAS 9.4©, Cary, NC.^23^ Sixty-one responses were missing on the dependent variables (3.1%) for the following reasons: 37 respondents selected both PA options; 13 selected none of the 24 items; and 11 selected “None of the above.” Due to the small percentage (<5%) missing on the dependent variables, data were treated with the default pairwise deletion, which uses all valid pairs of data and provides valid inference if data are missing at random (MAR) or missing completely at random (MCAR). Independent variables included income, a known sensitive item with a higher percent of non-response. However, this would be considered MAR data for the dependent variables (i.e., data are missing due to a predictor variable, not because of the outcome). The primary concern for reduction in sample size for the final logistic regression model (from n = 1951 to n = 1401, 28.2%) would be power, which was maintained for the effects found in this analysis.

Descriptive statistics included frequencies and valid percentages for all categorical variables. Logistic regression was used to model odds ratios (ORs) with 95% confidence limits (CLs) for each independent variable separately, and adjusted odds ratios (AORs) with 95% CLs for each independent variable when adjusted for all other independent variables. Data were assessed for violation(s) of the assumptions of logistic regression. Ethnicity was removed as an independent variable for violating the assumption of sufficient cell size.

## RESULTS

A total of 1,951 surveys met the sampling frame criteria, including 1,765 from H1 and 186 from H2. The majority of the respondents were White (n = 1,872, 97.6%), female (n = 1,289, 67.3%), married or in a domestic partnership (n = 1,323, 68.5%), residing in a mostly urban county (n = 1,425, 73.0%), and had good or excellent self-reported health (n = 1,155, 59.5%). Almost half of the respondents were classified as obese (n = 899, 49.0%). Compared with the county profiles (Table 1), survey respondents tended to be older, have more education and higher income, and over-represented in comparison to females.

As detailed in Table 2, roughly twice as many respondents indicated they spent less time doing PA during the pandemic (n = 584, 30.1%) than more time doing PA (n = 303, 16.0%). Over two-fifths (n = 323, 41.0%) of individuals with self-rated health of very poor, poor, or fair indicated spending less time doing PA during the pandemic. Only two subcategories of respondents more frequently indicated they spent more time doing PA than spent less time doing PA during the pandemic: (1) those reporting under- or normal weight (27.2% v. 20.4%) and (2) those with annual household income ≥$75,000 (24.5% v. 24.1%). A full description of the sample is provided in Table 2.

Table 3 displays the logistic regression results. More time spent doing PA during the pandemic was significantly positively associated with good/excellent self-rated health (AOR: 2.36, CL: 1.64, 3.39), under- or normal weight (AOR: 2.33; CL: 1.59, 3.42), overweight (AOR: 1.56; CL: 1.09, 2.22), having an annual household income of $75,000 or more (AOR: 2.48, CL: 1.40, 4.40), and having a bachelor’s degree or higher (AOR: 1.50, CL: 1.01, 2.25). Similarly, good/excellent self-rated health (AOR: 0.51, CL: 0.40, 0.65), under- or normal weight (AOR: 0.61, CL: 0.43, 0.86), having an annual household income of $75,000 or more (AOR: 0.59, CL: 0.39, 0.88), and male sex (AOR: 0.76, CL: 0.58, 0.99) were significantly associated with less time spent doing PA during the pandemic. That is, respondents in the referent group (i.e., very poor/poor/fair self-rated health, obese, <$30,000 annual household income, female) were more likely to decrease their time spent doing PA. Less time spent doing PA during the pandemic was also positively associated with a mostly urban county of residence (AOR: 1.57, CL: 1.01, 2.44).

## DISCUSSION

The survey data reported herein provide an initial understanding of the sociodemographic factors associated with PA increases and PA decreases during the COVID-19 pandemic among a sample of adults in Appalachia. Assessed in early 2021, results highlight the ongoing negative implications of the pandemic that reinforces and extends the findings of other studies conducted earlier in the pandemic in other areas of the U.S. Other studies conducted in March and June 2020, using similar items directly asking if PA increased, decreased, or stayed the same, revealed a similar percentage of respondents decreasing PA (30.4%) but a greater percentage increasing PA (20.3%)^11^ than observed here. Knell et al.^10^ reported a higher rate of both decreasing PA (39.0%) and increasing PA (25.2%), with surveys conducted in April/May 2020 using a continuous scale of PA. These findings are critical because lower levels of PA during the COVID-19 pandemic have been associated with negative mental health outcomes in adults,^24^ older adults,^7^ and college students.^25^–^27^ Further, Appalachian adults had higher prevalence of sedentarism (i.e., lack of leisure-time PA) prior to the pandemic.^1^ Decreasing the already low levels of PA in Appalachia, as the findings suggest is happening, could exacerbate the significant mental and physical health disparities in Appalachia that predated the pandemic.^1^ Significant investments of human, organizational, and fiscal capital; considering PA in all policy decisions; and increasing access to PA for Appalachian residents of all ages and abilities are potential solutions to sedentarism and ill health before they become endemic to the region.

In the adjusted models, factors associated with spending less time doing PA included living in a more urban area; being female; having obesity; having a lower household income; and reporting very poor, poor, or fair health. These populations should be prioritized for future interventions in Appalachia. The findings support those of Dunton et al.^9^, who found greater decreases in self-reported minutes of walking for respondents with low income, and those of Watson et al.^11^, who found that urban residence was associated with less PA during the pandemic. It’s likely that urban residents depended more on indoor PA at fitness facilities that were shuttered during the pandemic and/or had limited access to outdoor places for PA. Rural residents may also have been subject to fewer, shorter, and/or less stringently enforced COVID-19-related restriction policies, a factor associated with PA.^12^ It should be noted that (1) our county-level definition of rural differed from that used in similar research by Watson et al.^11^ and (2) the county where our mostly urban respondents resided had 26.8% of residents living outside an urban area, higher than national data (11.0%).^28^

As in Watson et al.^11^, in this study’s adjusted models, spending more time doing PA during the pandemic was associated with not having obesity and with having earned a bachelor’s degree or higher, though the Appalachian Region has higher obesity rates and a lower rate of bachelor’s degree attainment than the rest of the U.S.^1^ In addition, having greater self-rated health and having a higher annual household income were associated with more time spent doing PA during the pandemic, likely widening pre-pandemic health disparities that have been shown to be predictive of pandemic-related health behaviors in the U.K.^29^ and in the U.S.^12^

These findings do not support previous studies that found that women were more likely than men to increase PA during the pandemic^10^,^11^, nor did they concur with Knell et al.’s^10^ finding that having a child in the home was associated with increasing PA. Age, urbanicity and race were not significantly associated with increasing PA as they were in Watson et al.^11^, though our sample was older, and very homogeneous with respect to race, ethnicity, and geography, and these analyses adjusted for predictors.

### Limitations

The results of this study should be considered in light of multiple limitations. First, these data were collected using a convenience sample from one state that may not have accurately represented the population, limiting generalizability but complementing other regional survey research on COVID-19-related PA changes among adults in the U.S.^9^,^10^ Second, the dependent variables were assessed using a multiselect item similar to other COVID-19-related studies^18^,^19^ though without a “no change” response option, rendering an inability to identify true “no” selections. This was addressed through use of very conservative definitions to remove perceived non-responses. Third, the county-level definition of rurality utilized here was selected because the narrowest geography captured by the survey was county of residence; this limits comparability of study results with other relevant studies, and possibly overrepresents rural residents. Fourth, data collection occurred in winter months (January–March) which may have biased the responses about PA despite survey instructions asking respondents to consider “the past six months” and “in response to the coronavirus pandemic” when considering if they had increased and/or decreased time spent doing PA. The length of time of recall from the start of the pandemic to the survey data collection (9–12 months) may have also biased the responses.

## IMPLICATIONS

Despite limitations of the study, the results indicate ongoing PA reductions in a WV population in North Central Appalachia already experiencing significant health disparities. Future studies evaluating the impacts of the pandemic on PA and other key health behaviors should be conducted to advance understanding of the long-term effects, ideally using a longitudinal design and/or objective data.^9^,^13^,^30^ Our understanding of pandemic-related individual- and community-level facilitators of and barriers to PA should be further developed and utilized to evaluate methods to counteract any negative effects on key health behaviors, especially in health-disparate populations such as the one represented in this study.

**SUMMARY BOX**

**What is already known about this topic?**

In the U.S., a greater percentage of adults decreased than increased physical activity during the early months of the COVID-19 pandemic. Physical activity changes vary by demographic and social determinants of health.

**What is added by this report?**

Respondents to a community health needs survey from a convenience sample of adults in Appalachia had much higher rates of reporting decreases to physical activity than increases during the COVID-19 pandemic, nearly 9–12 months after the pandemic began. Survey respondents from completely rural counties were less likely than respondents from mostly urban counties to decrease PA, however.

**What are the implications for future research?**

Identifying the reasons for decreasing PA is now critical for public health practice because these decreases may be exacerbating pre-pandemic health disparities and so that these decreases can be addressed when we emerge more fully from the COVID-19 pandemic.

## Figures and Tables

**Table 1:** Census profile of counties where CHNA survey data were collected, 2019

County	Population	Rural Pop. (%)*	White (%)	Female (%)	Age, 65+ years (%)	Education, Bachelor’s degree or higher (%)†	Married (%)§	Households at least one person aged <18 years (%)	Median household income, (%>$75k)
Calhoun	7,295	100.0	99.0	51.7	23.5	12.2	57.4	25.3	38,382, (18.8%)
Jackson	28,907	71.4	97.6	50.4	19.9	17.4	56.0	26.4	47,837 (29.9%)
Pleasants	7,482	54.5	96.2	45.9	18.6	11.6	52.7	36.4	56,838 (37.0%)
Ritchie	9,844	100.0	97.4	50.4	22.9	11.3	55.4	26.7	43,577 (24.2%)
Roane	14,020	80.0	97.9	50.6	21.3	13.1	52.4	24.3	37,373 (24.4%)
Tyler	8,811	91.1	97.6	50.1	21.8	14.1	50.4	22.5	43,087 (24.8%)
Wirt	5,798	100.0	99.4	49.4	19.6	11.2	62.6	25.1	46,048 (20.4%)
Wood	84,960	26.8	96.2	51.7	19.9	21.9	49.7	27.7	47,321 (30.2%)

NOTES: Data are from the U.S. Census Bureau’s American Community Survey ((https://www.census.gov/programs-surveys/acs), 5-year estimates (2019) except for the rural population percentage (2010 Decennial Census; https://data.census.gov/cedsci/)

* Rural population percentage is the percentage of a county’s population not living in an urban area, 2010

† Education is educational attainment for population 25 years and older

§ Married is “now married (except separated)”, aged 15 years & older

**Table 2:** Descriptive statistics of survey respondents (n = 1,951) and COVID-19-related PA changes

Variable	All (n = 1951)		Yes, “Spent more time doing PA” (n = 303, 16.0%)*		Yes, “Spent less time doing PA” (n = 584, 30.1%)†	
		
	n	%§	n	%¶	n	%¶
County of residence	1951		303		584	

Mostly Urban	1425	73.0	218	15.3	436	30.6

Mostly Rural	347	17.8	64	18.4	108	31.1

Completely Rural	179	9.2	21	11.7	40	22.3

Age group	1947		303		584	

18–44	295	15.2	56	19.0	93	31.5

45–64	763	39.2	114	14.9	220	28.8

≥65	889	45.7	133	15.0	271	30.5

Self-rated health	1942		302		582	

Very poor/poor/fair	787	40.5	56	7.1	323	41.0

Good/ Excellent	1155	59.5	246	21.3	259	22.4

BMI	1836		293		546	

Underweight/Normal	367	20.0	100	27.2	75	20.4

Overweight	570	31.1	101	17.7	162	28.4

Obese	899	49.0	92	10.2	309	34.4

Gender	1916		300		578	

Male	627	32.7	100	15.9	167	26.6

Female	1289	67.3	200	15.5	411	31.9

Marital status	1932		303		581	

Other arrangements	609	31.5	68	11.2	211	34.6

Married/ in a domestic partnership	1323	68.5	235	17.8	370	28.0

Ethnicity	1878		296		564	

No, not Hispanic or Latino	1868	99.5	294	15.7	562	30.1

Yes, Hispanic or Latino	10	0.5	2	20.0	2	20.0

Race	1919		301			579

All other races	47	2.5	7	14.9	16	34.0

White	1872	97.6	294	15.7	563	30.1

Yearly household income	1593		251		487	

< $30,000	386	24.2	31	8.0	149	38.6

$30,000 to $49,999	350	22.0	43	12.3	116	33.1

$50,000 to $74,999	359	22.5	55	15.3	102	28.4

≥ $75,000	498	31.3	122	24.5	120	24.1

Education 1915		298		577		

≤ High school diploma or equivalent (GED)	638	33.3	65	10.2	198	31.0

Completed some post-secondary education	477	24.9	66	13.8	145	30.4

Bachelor’s degree or higher	800	41.8	167	20.9	234	29.3

Children in the home	1840		286		556	

None	1503	81.7	225	15.0	454	30.2

One or more	337	18.3	61	18.1	102	30.3

NOTES:

* Variable derived from 24-item checklist, coded as “yes” if respondents selected “Spent more time doing physical activity”; denominator is 1,890 after excluding 61 as “missing”

† Variable derived from 24-item checklist, coded as “yes” if respondents selected “Spent less time doing physical activity”; denominator is 1,890 after excluding 61 as “missing”

§ Column percentages presented out of the total responses for each variable (e.g., 1,915 for Education)

¶ Row percentages presented based on n reported in the “All” column for that row; row *n*s vary based on response to each item.

**Table 3:** Logistic regression, odds ratio, adjusted odds ratio (AOR), and AOR 95% confidence limits for factors associated with more time or less time spent doing PA during the COVID-19 pandemic (n = 1,401)

Predictor Variable	“Spent more time doing PA” (n = 229; 16.3%)			“Spent less time doing PA” (n = 432; 30.8%)		
	
	OR§	AOR	95% CL	OR§	AOR	95% CL
County of residence						

Mostly Urban	1.39	1.15	0.64–2.04	1.58†	1.57†	1.01–2.44

Mostly Rural	1.70†	1.53	0.81–2.90	1.57†	1.40	0.85–2.31

Completely Rural	1 [Reference]			1 [Reference]		

Age group						

18–44	1.36	1.00	0.60–1.67	1.07	1.11	0.75–1.65

45–64	1.00	0.89	0.63–1.27	0.92	0.88	0.67–1.16

≥65	1 [Reference]			1 [Reference]		

Self-rated health						

Good/ Excellent	3.56*	2.36*	1.64–3.39	0.41*	0.51*	0.40–0.65

Very poor/poor/fair	1 [Reference]			1 [Reference]		

BMI						

Underweight/Normal	3.35*	2.33*	1.59–3.42	0.49*	0.61*	0.43–0.86

Overweight	1.9*	1.56*	1.09–2.22	0.76†	0.92	0.70–1.20

Obese	1 [Reference]			1 [Reference]		

Sex						

Male	1.04	0.88	0.63–1.23	0.78†	0.76†	0.58–0.99

Female	1 [Reference]			1 [Reference]		

Marital Status						

Married/ in a domestic partnership	1.70*	0.96	0.65–1.42	0.72*	1.05	0.78–1.41

Other arrangements	1 [Reference]			1 [Reference]		

Race						

White	1.04	0.97	0.32–2.92	0.81	0.98	0.45–2.14

All other races	1 [Reference]			1 [Reference]		

Yearly household income						

≥ $75,000	3.77*	2.48*	1.40–4.40	0.51*	0.59*	0.39–0.88

$50,000 to $74,999	2.09*	1.58	0.90–2.77	0.63*	0.70	0.48–1.02

$30,000 to $49,999	1.62†	1.51	0.87–2.60	0.79	0.86	0.60–1.22

< $30,000	1 [Reference]			1 [Reference]		

Education						

Bachelor’s degree or higher	2.30*	1.50†	1.01–2.25	0.90	1.16	0.86–1.57

Completed some post-secondary education	1.41	1.22	0.78–1.91	0.96	1.10	0.81–1.51

≤ High school diploma or equivalent (GED)	1 [Reference]			1 [Reference]		

Children in home						

None	0.78	0.96	0.61–1.51	0.97	0.90	0.63–1.28

One or more	1 [Reference]			1 [Reference]		

**Model fit information**						

R^2^	0.13			0.07		

AIC	1168.47			1690.54		

NOTES: AOR = odds ratio adjusted for all other predictor variables listed in the table, Ref = reference group, R^2^ = Pseudo max-rescaled, AIC = Akaike Information Criteria, BMI = body mass index, PA = physical activity.

* *p* ≤ .01

† *p* ≤ .05

§ “n” is different for unadjusted OR than the AOR models due to pairwise deletion method

## References

[1] Marshall JL Thomas L Lane NM Holmes GM Arcury TA Randolph R Health disparities in Appalachia Washington (DC) Appalachian Regional Commission 2017 Available from: https://www.arc.gov/report/health-disparities-in-appalachia/ Accessed Apr. 8, 2022

[2] Booth FW Gordon SE Carlson CJ Hamilton MT Waging war on modern chronic diseases: primary prevention through exercise biology J Appl Physiol 2000 88 2 774 87 10.1152/jappl.2000.88.2.774 10658050

[3] Booth F Chakravarthy M Cost and consequences of sedentary living: new battleground for an old enemy Washington (DC) President’s Council on Physical Fitness and Sports Research Digest 2002 3 16 1 8

[4] 2018 Physical Activity Guidelines Advisory Committee 2018 physical activity guidelines advisory committee scientific report Washington (DC) United States Department of Health and Human Services (USDHHS) 2018

[5] Sallis R Young DR Tartof SY Sallis JF Sall J Li Q Physical inactivity is associated with a higher risk for severe COVID-19 outcomes: a study in 48,440 adult patients Br J Sports Med 2021 55 19 1099 10.1136/bjsports-2021-104080 33849909

[6] Meyer SM Landry MJ Gustat J Lemon SC Webster CA Physical distancing ≠‚ physical inactivity Transl Behav Med 2021 11 4 941 4 10.1093/tbm/ibaa134 33410492 PMC7928669

[7] Creese B Khan Z Henley W O’Dwyer S Corbett A Da Silva MV Loneliness, physical activity, and mental health during COVID-19: a longitudinal analysis of depression and anxiety in adults over the age of 50 between 2015 and 2020 Int Psychogeriatr 2021 33 5 505 14 10.1017/S1041610220004135 33327988 PMC7985900

[8] Ginoux C Isoard-Gautheur S Teran-Escobar C Forestier C Chalabaev A Clavel A Being active during the lockdown: the recovery potential of physical activity for well-being Int J Environ Res Public Health 2021 18 4 1707 10.3390/ijerph18041707 33578869 PMC7916567

[9] Dunton GF Wang SD Do B Courtney J Early effects of the COVID-19 pandemic on physical activity locations and behaviors in adults living in the United States Prev Med Rep 2020 20 101241 10.1016/j.pmedr.2020.101241 33173751 PMC7644187

[10] Knell G Robertson MC Dooley EE Burford K Mendez KS Health behavior changes during COVID-19 pandemic and subsequent “stay-at-home” orders Int J Environ Res Public Health 2020 17 17 6268 10.3390/ijerph17176268 32872179 PMC7504386

[11] Watson KB Whitfield GP Huntzicker G Omura JD Ussery E Chen TJ Cross-sectional study of changes in physical activity behavior during the COVID-19 pandemic among US adults Int J Behav Nutr Phys Act 2021 18 1 91 10.1186/s12966-021-01161-4 34233691 PMC8261396

[12] Wijngaards I del Pozo Cruz B Gebel K Ding D Exercise frequency during the COVID-19 pandemic: A longitudinal probability survey of the US population Prev Med Rep 2022 25 101680 10.1016/j.pmedr.2021.101680 34976708 PMC8710431

[13] Tison GH Avram R Kuhar P Abreau S Marcus GM Pletcher MJ Worldwide effect of COVID-19 on physical activity: a descriptive study Ann Intern Med 2020 173 9 767 70 10.7326/M20-2665 32598162 PMC7384265

[14] Whitfield GP Carlson SA Ussery EN Fulton JE Galuska DA Petersen R Trends in meeting physical activity guidelines among urban and rural dwelling adults — United States, 2008–2017 MMWR Morb Mortal Wkly Rep 2019 68 23 513 8 10.15585/mmwr.mm6823a1 31194722 PMC6613551

[15] Whitfield GP Carlson SA Ussery EN Watson KB Berrigan D Fulton JE National-level environmental perceptions and walking among urban and rural residents: informing surveillance of walkability Prev Med 2019 123 101 8 10.1016/j.ypmed.2019.03.019 30878571 PMC10885855

[16] Sallis JF Slymen DJ Conway TL Frank LD Saelens BE Cain K Income disparities in perceived neighborhood built and social environment attributes Health Place 2011 17 6 1274 83 10.1016/j.healthplace.2011.02.006 21885324

[17] LLC Qualtrics Qualtrics XM © Provo, UT, USA 2020

[18] National Institutes of Health All of Us research program COVID-19 Participant Experience (COPE) survey Available from: https://www.researchallofus.org/data-tools/survey-explorer/cope-survey/

[19] NORC at the University of Chicago COVID Impact Survey, conducted by NORC the University of Chicago for the Data Foundation Available from: https://www.covid-impact.org

[20] Office of the Governor West Virginia’s response to COVID-19 Available from: https://governor.wv.gov/Pages/WV-COVID-19-actions-and-executive-orders.aspx Accessed Apr. 18, 2023

[21] United States Census Bureau 2010 Census Urban and Rural Classification and Urban Area Criteria Available from: https://www.census.gov/geo/reference/ua/urban-rural-2010.html

[22] Abildso CG Daily SM Umstattd Meyer MR Edwards MB Jacobs L McClendon M Perry CK Roemmich JN Environmental factors associated with physical activity in rural U.S. counties Int J Environ Res Public Health 2021 18 14 10.3390/ijerph18147688 PMC8307667 34300138

[23] SAS Institute SAS 9.4© 2013

[24] Young DR Hong BD Lo T Inzhakova G Cohen DA Sidell MA The longitudinal associations of physical activity, time spent outdoors in nature and symptoms of depression and anxiety during COVID-19 quarantine and social distancing in the United States Prev Med 2022 154 106863 10.1016/j.ypmed.2021.106863 34774881 PMC8717103

[25] Huckins JF daSilva AW Wang W Hedlund E Rogers C Nepal SK Mental health and behavior of college students during the early phases of the COVID-19 pandemic: longitudinal smartphone and ecological momentary assessment study J Med Internet Res 2020 22 6 e20185 10.2196/20185 32519963 PMC7301687

[26] Mack DL DaSilva AW Rogers C Hedlund E Murphy EI Vojdanovski V Mental health and behavior of college students during the COVID-19 pandemic: longitudinal mobile smartphone and ecological momentary assessment study, part II J Med Internet Res 2021 23 6 e28892 10.2196/28892 33900935 PMC8183598

[27] Wilson OWA Holland KE Elliott LD Duffey M Bopp M The impact of the COVID-19 pandemic on us college students’ physical activity and mental health J Phys Act Health 2021 18 3 272 8 10.1123/jpah.2020-0325 33601332

[28] Ratcliffe M Burd C Holder K Fields A Defining rural at the US Census Bureau Washington (DC) United States Census Bureau 2016 8 Report No.: ACSGEO-1

[29] Naughton F Ward E Khondoker M Belderson P Marie Minihane A Dainty J Health behaviour change during the UK COVID-19 lockdown: findings from the first wave of the C-19 health behaviour and well-being daily tracker study Br J Health Psychol 2021 26 2 624 43 10.1111/bjhp.12500 33410229 PMC9291054

[30] Bourdas DI Zacharakis ED Evolution of changes in physical activity over lockdown time: physical activity datasets of four independent adult sample groups corresponding to each of the last four of the six COVID-19 lockdown weeks in Greece Data Brief 2020 32 106301 10.1016/j.dib.2020.106301 32934972 PMC7483084

